# Novel coronavirus and regular physical activity involvement: Opinion

**DOI:** 10.4102/phcfm.v12i1.2453

**Published:** 2020-05-21

**Authors:** Sunday O. Onagbiye, Zandile J.R. Mchiza, Susan H. Bassett, Andre Travill, Bert O. Eijnde

**Affiliations:** 1 Department of Sport, Recreation and Exercise Science, Faculty of Community and Health Science, University of the Western Cape, Cape Town, South Africa; 2 School of Public Health, Faculty of Community and Health Science, University of the Western Cape, Cape Town, South Africa; 3 SMRC Sports Medical Research Center and BIOMED Biomedical Research Institute, Faculty of Medicine and Life Sciences, Hasselt University, Diepenbeek, Belgium

**Keywords:** NCOVID-19, physical exercise, respiratory difficulties, well-being, SSA

## Abstract

The novel coronavirus (NCOVID-19) has quickly become a public health concern globally and needs urgent attention. While there is no current evidence of vaccines and specific drugs to prevent and treat the ailments emanating from NCOVID-19 infections, complementary and conventional medical treatments could prove beneficial in ameliorating some of the respiratory difficulties, especially in countries in sub-Saharan Africa. These treatments include specific breathing exercises, a diet that strengthens the immune system, as well as avoiding tobacco smoking and alcohol consumption. On the other hand, for those who have not contracted the virus, participation in indoor and within-the-yard physical activity could be beneficial in preventing unwanted weight gain as well as associated conditions such as anxiety and depression.

The novel coronavirus (NCOVID-19) has quickly become a public health concern globally and needs urgent attention. Novel coronavirus is a respiratory disease that has been branded by the World Health Organization^1^ as a new type of coronavirus (CoV) termed as severe acute respiratory syndrome (SARS) CoV 2 (SARS-CoV-2) that can cause a respiratory tract infection to both upper (i.e. sinuses, nose and throat) and, or lower (i.e. windpipe and lungs) respiratory tracts.^2^ Coronaviruses are a large family of viruses that cause respiratory illnesses that range from common cold to more severe lung diseases.^2,3^ These viruses include Middle East respiratory syndrome (MERS) and SARS.^3^ While the infections because of NCOVID-19 have been slow to reach Africa, there is a great concern that the recent growth in infections will further deteriorate the already struggling health and economic systems on the continent.

The NCOVID-19 cases in Africa as of 21 April 2020 stand at 23 517, with the highest number of cases (3300) reported in South Africa.^4^ However, these day-to-day estimations are anticipated to upsurge.^5^ In sub-Saharan Africa (SSA), for instance, the healthcare system faces major challenges. This includes insufficient funding, poor infrastructure, inequities, lack of health and scientific experts and medical personnel, and the brain drain of home-grown health and scientific experts relocating overseas for better remuneration and quality of life.^6^

Given the afore-mentioned health challenges that have currently compounded the existing problems in the healthcare system in this region, many countries (including South Africa in the SSA region) undertook safety precautions to protect its citizens from this outbreak.^5^ Among these precautions, many SSA countries have mandated ‘country lockdown’, coupled with basic protective and safety measures, against NCOVID-19, commissioned by the World Health Organization.^7^ Lockdown in this context could be understood as a restriction of people to their homes, typically to regain control over, or curb the spread of, the NCOVID-19 pandemic. However, the impact of the lockdown is feared to have dire consequences to the ailing economic systems of the countries, as well as the physical and psychological outcomes of their citizenry.

For instance, the *lockdown* has been described as *war-time* against NCOVID-19, which is also termed as the ‘invisible enemy’. This lockdown mandates social distancing and or solitary isolation or confinement. Moreover, it has caused restrictions on citizen’s usual daily routine.^1^ For instance, bans have been put in place to stop both national and international travel and sales of some goods, also prohibiting outdoor activities such as jogging or walking dogs.^8^ It is hoped that adhering to the restrictions and bans could flatten the NCOVID-19 infection curve. However, this lockdown could also impact the citizens’ physical and psychological well-being, especially in countries where the majority of citizens grapple with poverty, informal housing with restricted liveable space and lack of access to basic services. After the end of the lockdown cycles, countries (especially SSA countries) could see increases in the prevalence of overweight and obesity and their associated consequences, as well as an upsurge in cases of anxiety and depression.

As such, participation in indoor and within-the-yard physical activity and or exercise could be beneficial in ameliorating some of the respiratory difficulties, weight gain, as well as depression and psychological distress. After all, substantiated evidence globally^9,10,11^ supports this statement. Most importantly, it has been reported that physical activity and exercise can protect against some of the symptoms of respiratory infections^12^ and thereby lower inflammation and improve the immune reaction to respiratory viral infections.^13^ There is strong evidence^14^ to suggest that the immune system is responsive to physical exercise. However, exercise immunologists describe this response to be duration- and intensity-dependent. Nieman^15^ has for long shown that the relationship between infection risk and the extent of exercise follows a J-curve. This means that doing regular moderate exercise for a short period lowers one’s risk of infections compared to doing no form of physical activity. However, ramping the dose of activity or exercise up too high, and doing it over a long period, increases the risk of infections steadily until one becomes more vulnerable than if they were inactive in the first place.^15^

Given the afore-mentioned evidence suggesting lengthy and extreme physical exercise to trigger immune inhibition is unwarranted. Instead, short duration and moderate-intensity exercise seems to be ideal in improving immune function, as it can lower the risks and difficulties in breathing that are brought about by viral infection.^13^ It is also known that respiratory exercise and moderate physical activity that lasts for at least 30 min per day could help with breathing difficulties.^13,16^ Also, as aerobic exercise (in the presence of oxygen) enhances heart function and fortifies muscle contraction, breathing exercise could improve the effectiveness of the lungs as a means of rehabilitation, especially for those individuals with chronic obstructive pulmonary disease (COPD).^17^

Furthermore, regular exercise could protect against respiratory infections such as influenza, runny nose and dry cough.^12^ However, a study that evaluated the effectiveness of an exercise training programme on cardiorespiratory and musculoskeletal performance, as well as health-related quality of life of patients who were recovering from SARS, revealed that the programme was effective in improving both cardiorespiratory and musculoskeletal health.^18^ Therefore, regular, short-term, moderate-intensity physical exercise that can be performed indoors, or within the home environment, seems the best option for all those who are affected by the country lockdown regulations.

Promoting physical activity and exercise during lockdown should be a priority for the entire family. Moderate physical exercises that can be performed within the home space, such as dancing, gardening and house chores, should also be prioritised because these activities do not require ‘sophisticated’ equipment. Partnerships between the Department of Health and non-governmental organisations could help create awareness about novel health-promoting physical activity and exercise via the media (i.e. television, radio and social media). For instance, they could promote the foundation for Global Community Health (GCH) online physical activity and exercise programme known as Brain Breaks® (BB) (https://brain-breaks.com/)^19^ as a strategy that can be used at home because it is now available for free online worldwide. Brain Breaks® are short, web-based, 3–5-min games and videos aimed at getting all individuals moving and bringing about alertness and motivation to generate a healthier sphere. This will be beneficial to communities, especially those who may be at high risk of COVID-19 infections or those who may have pre-existing conditions. The approach of involving both the government and non-governmental organisations in advertising a physical activity programme like BB® could lead to some level of encouragement for all citizens to participate in health-enhancing moderate physical activity. After all, recent developments point to the fact that many people in Africa can afford to download this programme, given that 525 million people have access to the Internet.^20^ This could help people of all ages achieve and, or maintain good health and better their quality of life, especially during the country lockdown.

In an effort to flatten the infection curve and reduce the spread of NCOVID-19, ‘stay at home’ regulations, coupled with healthy eating and tackling physical inactivity, should be a priority for individuals of all age groups. Consumption of a balanced diet that strengthens the immune system and engaging in regular physical exercise has the potential to guard against respiratory infections.^14^ These health strategies may also be protective against weight gain and psychological distress. Not being active, on the other hand, could expose individuals to heart attacks, type 2 diabetes, some forms of cancer, as well as weak bones and muscles. These health conditions when coupled with NCOVID-19 infection increase the chances of hospitalisation and death. These are unwanted health outcomes because they tend to burden the already strained health system, especially in SSA countries that are also grappling with economic depression.^5^ Hence, engagement in regular physical activity and exercise^21^ in the secured home environment coupled with other important health behaviours, such as the cessation of tobacco smoking^22,23^ and alcohol consumption,^24^ are seen as relevant targeted interventions during the NCOVID-19-motivated lockdown.
