# Corrigendum: Novel coronavirus and regular physical activity involvement: Opinion

**DOI:** 10.4102/phcfm.v13i1.3186

**Published:** 2021-12-09

**Authors:** Sunday O. Onagbiye, Zandile J.R. Mchiza, Susan H. Bassett, Andre Travill, Bert O. Eijnde

**Affiliations:** 1 Department of Sport, Recreation and Exercise Science, Faculty of Community and Health Science, University of the Western Cape, Cape Town, South Africa; 2 School of Public Health, Faculty of Community and Health Science, University of the Western Cape, Cape Town, South Africa; 3 SMRC Sports Medical Research Center and BIOMED Biomedical Research Institute, Faculty of Medicine and Life Sciences, Hasselt University, Diepenbeek, Belgium

In the version of this article initially published, Onagbiye SO, Mchiza ZJR, Bassett SH, Travill A, Eijnde BO. Novel coronavirus and regular physical activity involvement: Opinion. Afr J Prm Health Care Fam Med. 2020;12(1), a2453. https://doi.org/10.4102/phcfm.v12i1.2453, reference number 5 on page 3 was given incorrectly. The correct reference should be ‘Chen P, Mao L, Nassis GP, Harmer P, Ainsworth BE, Li F. Coronavirus disease (COVID-19): The need to maintain regular physical activity while taking precautions. J Sport Health Sci. 2020 Mar;9(2):103–104. https://doi.org/10.1016/j.jshs.2020.02.001’ instead of ‘Chen P, Mao L, Nassis GP, Harmer P, Ainsworth BE, Li F. Wuhan coronavirus (2019-nCoV): The need to maintain regular physical activity while taking precautions. J Sport Health Sci. 2020;9(2):103. https://doi.org/10.1016/j.jshs.2020.02.001’ in the ‘References’ section.

This correction does not alter the study’s findings of significance or overall interpretation of the study’s results. The authors apologise for any inconvenience caused.

